# Supplementary material for: Design automation of microfluidic single and double emulsion droplets with machine learning
Source: Nat Commun. 2024 Jan 2;15:83. doi: 10.1038/s41467-023-44068-3 (PMC10761910; doi:10.1038/s41467-023-44068-3)
Supplement: Supplementary file 1 — Supplementary information [file 41467_2023_44068_MOESM1_ESM.pdf]

Design Automation of Microfluidic Single and Double Emulsion Droplets with Machine Learning

Ali Lashkaripour, David P. McIntyre, Suzanne G.K. Calhoun, Karl Krauth, Douglas M. Densmore, and Polly M. Fordyce

November 29, 2023

Supplementary Material

1 Supplementary Note 1: Literature scaling laws

To establish the baseline for performance prediction accuracy, we fitted a number of scaling laws from the literature to our comprehensive dataset and assessed their accuracy in predicting droplet diameter and generation rate.

First, we considered the Fu et al. scaling law [2], where a dimensionless droplet size (i.e., droplet size normalized by the oil inlet width) is proposed to scale with flow rate ratio and capillary number, as given in Eq. (1):

$$\frac{L}{W_{or}} = c_1(\frac{Q_d}{Q_c})^{c_2} Ca_c^{c_3} \tag{1}$$

Here, L is droplet size, W_{or} is the orifice width, Q_d is the flow rate of the dispersed fluid, Q_c is the flow rate of the continuous fluid, and Ca_c is the capillary number of the continuous fluid. This model showed a MAPE of 21.7% in predicting the droplet diameter and 88.9% in predicting generation rate of the 20% test set in the comprehensive dataset.

We next attempted to improve the Fu et al. scaling law by normalizing the droplet diameter with the hydraulic diameter instead of the orifice width, as given in Eq. (2).

$$\frac{L}{D_{hyd}} = c_1(\frac{Q_d}{Q_c})^{c_2} Ca_c^{c_3} \tag{2}$$

This improved model predicted droplet diameter with a MAPE of 17.2% and generation rate with a MAPE of 78.6% for the test set of the comprehensive dataset, with an example run shown in Fig. S.3.

Next, we considered the Liu et al. scaling law [5], where droplet diameter normalized by the oil inlet width scales with a capillary number and flow rate ratio as given in Eq. (4):

$$\frac{D}{W_c} = (c_1 + C_2\frac{Q_d}{Q_c}) Ca^{c_3} \tag{3}$$

This scaling law was not accurate and resulted in a MAPE of 47.6% in predicting droplet size and a MAPE of 3023% in predicting the generation rates of the test set, as shown in Fig. S.(4).

Additionally, we considered an improved version of the Liu et al. scaling law [5] in which the droplet diameter is normalized by orifice width instead of the oil inlet width, as given in Eq. (4).

$$\frac{D}{W_{or}} = (c_1 + C_2\frac{Q_d}{Q_c}) Ca^{c_3} \tag{4}$$

The improved version of the Liu et al. scaling law showed a MAPE of 24.4% and 100.7% in predicting the droplet diameter and generation rate of the comprehensive test set, as shown in Fig. S5.

Next, we fitted a scaling law proposed by Xu et al. [8], where the normalized droplet diameter scales non-linearly with flow rate ratio and capillary number, as given in Eq. (5).

$$\frac{D}{W_{or}} = c_1 + c_2(\frac{Q_d}{Q_c})^{c_3} Ca^{c_4} \tag{5}$$

This scaling law predicted the droplet diameters and generation rates in the test set of the comprehensive dataset with a MAPE of 20.7% and 83.5%, respectively. An example test is shown in Fig. S. 6.

We then improved the scaling law proposed by Xu et al. [8] by normalizing the droplet diameter by the hydraulic diameter instead of the orifice width, as given in Eq. (6).

$$\frac{D}{D_{hyd}} = c_1 + c_2(\frac{Q_d}{Q_c})^{c_3} Ca^{c_4} \tag{6}$$

The improved scaling law was capable of predicting droplet diameter with a MAPE of 17.2% and generation rate with a MAPE of 78.5% within the test set of the comprehensive dataset, as shown in Fig. S.7.

Next, we considered a scaling law proposed by Lee et al. [4], where the droplet diameter normalized by the hydraulic diameter scales with capillary number, as given in Eq. 7.

$$\frac{D}{D_{hyd}} = c_1 Ca^{c_2} \tag{7}$$

This scaling law predicted the droplet diameters and generation rates in the test set of the comprehensive dataset with a MAPE OF 34.9% and 324%, respectively, as shown in Fig. S.8.

Next, we fitted a scaling law proposed by Ward et al. [7], where the droplet diameter normalized by the orifice scales with the ratio of dispersed and continuous flow rates, as given in Eq. (8).

$$\frac{D}{W_{or}} = c_1(\frac{Q_d}{Q_c})^{c_2} \tag{8}$$

This scaling law predicted the droplet diameter with a 21.3% MAPE and generation rate with a 139% MAPE for the datapoints in the test set of the comprehensive dataset, as shown in Fig. S.9.

Next, we fitted a scaling law proposed by Zheya Liu et al. [6] to our data. In this scaling law the normalized droplet diameter by the hydraulic diameter scales with capillary number, flow rate ratio, and viscosity ratio, as given in Eq. (9).

$$\frac{D}{D_{hyd}} = c_1(\frac{Q_d}{Q_c})^{c_2}(\frac{\lambda_d}{\lambda_c})^{c_3} Ca_c^{c_4} \tag{9}$$

This scaling law demonstrated the highest accuracy of all previously published scaling laws in predicting our comprehensive dataset, with a MAPE of 17.7% in predicting the diameter and 58.7% in predicting the generation rate, as shown in Fig. S.10.

Finally, we proposed a new scaling law that takes capillary number, flow rate ratio, and viscosity ratio to predict the droplet diameter normalized by the hydraulic diameter of the orifice, as given in Eq. 10.

$$\frac{D}{D_{hyd}} = c_1 + c_2(\frac{Q_d}{Q_c})^{c_3}(c_4 + Ca^{c_5})(\frac{\lambda_c}{\lambda_d})^{c_6} \tag{10}$$

The proposed scaling law was able to predict the droplet diameter with a MAPE of 13.6% and generation rate with a MAPE of 46.9%, as shown in Fig. S.11.

A single comparison of the MAPE in predicting droplet size and rate for all the scaling laws is depicted in Fig. S.12.

2 Supplementary Note 2: Effect of including viscosity ratio as an input of Neural Networks

Including viscosity ratio as the 8th input of the neural networks minutely improved their accuracy for predicting droplet diameters and generation rates of the comprehensive dataset (MAPE of 7.15% vs 7.45%). However, including this parameter reduced its accuracy in predicting literature data, as are provided in Supplementary Table 3.

3 Supplementary Note 3: Percentage error relationship between droplet diameter and generation rate prediction

For both machine learning models, the MAPE for generation rate was approximately 3 times the MAPE for diameter. This is mathematically expected according to conservation of mass, as generation rate inversely scales with the 3rd power of diameter.

$$F \sim \frac{1}{D^3} \tag{11}$$

For a small error in predicting diameter (δ_d), using Taylor series expansion we have:

$$\frac{1}{(D+\delta_d)^3} \approx \frac{1}{D^3} - \frac{3\delta_d}{D^4} + \mathrm{O}\left(\delta_d^2\right). \tag{12}$$

Therefore, prediction error for generation rate (δ_f) is approximated by:

$$\delta_f \sim \frac{1}{D^3} - \frac{1}{(D+\delta_d)^3} \approx \frac{3\delta_d}{D^4} - \mathrm{O}\left(\delta_d^2\right). \tag{13}$$

For small errors of δ_d, $\mathrm{O}\left(\delta_d^2\right)$ is negligible and δ_f is approximated by:

$$\delta_f \sim \frac{3\delta_d}{D^4}. \tag{14}$$

Percentage error in predicting generation rate can then be approximated by dividing Eq. 14 by Eq. 11. Therefore, for small values of δ_d, percentage error for generation rate (ε_f) is approximated by 3 times the percentage error for diameter (ε_d):

$$\varepsilon_f = \frac{\delta_f}{F} \approx \frac{3\delta_d}{D} = 3\varepsilon_d \tag{15}$$

Therefore, we expect rate MAPE of accurate models to be 3 times the MAPE of the predicted diameter [3].

4 Supplementary Note 4: Comparison of DAFD 3.0 and DAFD 1.0 in generalizability to as-yet-unseen fluids and geometries

We compared the accuracy of DAFD 3.0 and DAFD 1.0 in predicting droplet diameters for as-yet-unseen fluids and geometries included in the ”Machine learning models can generalize to previously unseen geometries and fluids” section of the main manuscript. DAFD 1.0 predicted these data with low accuracy and yielded a MAPE of 92.7% (Fig. S.21). However, as discussed in the main manuscript, the neural network, boosted decision trees, and consensus model were able to predict the diameters with an MAPE of 12.1%, 10.7%, and 10.1%, respectively. We next established that this improved generalizability is mainly due to the diversity of the new comprehensive dataset that includes several fluids, geometries, and device materials (Fig. S.22) and an improved model training approach namely a dimensionless output (normalized droplet diameter by the hydraulic diameter of the orifice), L2 regularization, and a shallow and wide neural network structure (Fig. S.22).

Supplementary Table 1: Comparison of previously published experimental datasets and machine learning models for predicting droplet diameter in microfluidic flow-focusing droplet generation in the dripping regime.

**Parameter**	Chagot et al. (2022) [1]	Lashkaripour et al. (2021) [3]	This study
Number of datapoints	468	474	868
Range of diameters	73 - 190 μm	25 - 250 μm	15 - 250 μm
Range of generation rates	5 - 800 Hz	5 - 500 Hz	5 - 12000 Hz
Variations in geometry	No	Yes	Yes
Variations in fluids	No	No	Yes
Variations in surfactants	Yes	No	Yes
Aqueous-in-oil droplets	Yes	Yes	Yes
Oil-in-aqueous droplets	No	No	Yes
Single/Double emulsions	Single	Single	Single & Double
Dimensionless inputs	No	Yes	Yes
Dimensionless output	Yes	No	Yes
Tool for performance prediction	No	Yes	Yes
Tool for design automation	No	Yes	Yes

Supplementary Table 2: Fitting constants for all the scaling laws tested against our comprehensive dataset on SE and DE droplet generation.

Scaling law	Fitting constants					
	c_1	c_2	c_3	c_4	c_5	c_6
$\frac{D}{D_{hyd}} = c_1 Ca^{c_2}$ [4]	0.590	-0.043	N/A	N/A	N/A	N/A
$\frac{D}{W_{or}} = c_1 (\frac{Q_d}{Q_c})^{c_2}$ [7]	1.579	0.373	N/A	N/A	N/A	N/A
$\frac{D}{W_{or}} = c_1 (\frac{Q_d}{Q_c})^{c_2} Ca_c^{c_3}$ [2]	1.566	0.347	-0.062	N/A	N/A	N/A
$\frac{D}{D_{hyd}} = c_1 (\frac{Q_d}{Q_c})^{c_2} Ca_c^{c_3}$	1.534	0.373	-0.009	N/A	N/A	N/A
$\frac{D}{W_c} = (c_1 + C_2 \frac{Q_d}{Q_c}) Ca^{c_3}$ [5]	0.162	0.447	-0.080	N/A	N/A	N/A
$\frac{D}{W_{or}} = (c_1 + C_2 \frac{Q_d}{Q_c}) Ca^{c_3}$	0.538	1.507	-0.047	N/A	N/A	N/A
$\frac{D}{W_{or}} = c_1 + c_2 (\frac{Q_d}{Q_c})^{c_3} Ca^{c_4}$ [8]	-6.687	8.183	0.043	-0.0066	N/A	N/A
$\frac{D}{D_{hyd}} = c_1 + c_2 (\frac{Q_d}{Q_c})^{c_3} Ca^{c_4}$	-0.303	1.784	0.273	-0.012	N/A	N/A
$\frac{D}{D_{hyd}} = c_1 (\frac{Q_d}{Q_c})^{c_2} (\frac{\lambda_d}{\lambda_c})^{c_3} Ca_c^{c_4}$ [6]	1.768	0.333	-0.105	-0.089	N/A	N/A
$\frac{D}{D_{hyd}} = c_1 + c_2 (\frac{Q_d}{Q_c})^{c_3} (c_4 + Ca^{c_5}) (\frac{\lambda_c}{\lambda_d})^{c_6}$	-0.173	0.010	0.293	189.93	1.313	-0.040

Supplementary Table 3: Effect of including viscosity ratio as the 8th input parameter of the neural network on its accuracy for predicting droplet diameter and generation rate in the comprehensive dataset and their generalizability to predicting droplet diameters from previously published data.

Parameter	MAPE	R^2	MAE	RMSE
**Comprehensive dataset droplet diameter prediction**				
Train set	5.56 ±0.22%	0.98 ±0	4.15 ±0.16μm	6.70 ±0.21μm
Test set	7.15 ±0.29%	0.95 ±0	5.64 ±0.29μm	9.36 ±0.43μm
**Comprehensive dataset generation rate prediction**				
Train set	16.73 ±0.34%	0.97 ±0	248 ±12.07Hz	476.97 ±16.75Hz
Test set	21.91 ±0.93%	0.97 ±0	262.84 ±12Hz	500.16 ±18.79Hz
**Literature data diameter prediction**				
Literature data	13.02 ±2.98%	0.82 ±0.03	15.63 ±5.46μm	24.25 ±9.70μm

Supplementary Table 4: DAFD 3.0 inputs and outputs for design automation of SEs of complete RPMI 1640 media with added 20% optiprep and 0.1% pluronic F-127 in dSurf HFE 7500, related to Fig. 6a of the main manuscript. The fluid properties of the dispersed and continuous fluids and the interfacial tension between them were inputted using the values provided in Table 1 of the main manuscript.

**Desired performance**			**Proposed designs**						
**Performance**		**Constraints**	**Geometry (μm)**					**Flow rates (μl/hr)**	
Diameter	Rate		Orifice	Depth	Outlet	Disp. inlet	Cont. inlet	Disp. fluid	Cont. fluid
25 μm	2000 Hz	geometry	22.5	30	22.5	22.5	22.5	64.1	392.3
30 μm	2000 Hz	geometry	22.5	30	22.5	22.5	22.5	95.3	392.3
35 μm	1000 Hz	geometry	22.5	30	22.5	22.5	22.5	75.5	158.8

Supplementary Table 5: DAFD 3.0 inputs and outputs for design automation of DEs using complete RPMI 1640 media with added 20% optiprep and 0.1% pluronic F-127, dSurf HFE 7500, and complete RPMI 1640 media with added 5% pluronic F-127 related to Fig. 6b of the main manuscript. The specified geometric constraints at FF2 were kept at 2x the same values at FF1 to facilitate stable DE generation. The fluid properties of the inner, middle, and outer fluids and the interfacial tensions between them were inputted using the values provided in Table 1 of the main manuscript.

**Desired performance**			**Proposed designs**							
**Performance**		**Constraints**	**Geometry at FF1 (μm)**					**Flow rates (μl/hr)**		
Inner diam.	Outer diam.		Orifice	Depth	Outlet	Disp. inlet	Cont. inlet	Inner	Middle	Outer
25 μm	50 μm	geometry	22.5	30	22.5	22.5	22.5	100	650	4000
30 μm	50 μm	geometry	22.5	30	22.5	22.5	22.5	100	375	2500
35 μm	50 μm	geometry	22.5	30	22.5	22.5	22.5	125	200	2000
40 μm	50 μm	geometry	22.5	30	22.5	22.5	22.5	525	400	5500
25 μm	55 μm	geometry	22.5	30	22.5	22.5	22.5	50	500	2500
30 μm	55 μm	geometry	22.5	30	22.5	22.5	22.5	50	225	1500
35 μm	55 μm	geometry	22.5	30	22.5	22.5	22.5	125	200	2000
40 μm	55 μm	geometry	22.5	30	22.5	22.5	22.5	525	400	5500
30 μm	45 μm	geometry	22.5	30	22.5	22.5	22.5	400	1075	9500

Supplementary Table 6: Design parameter range and step size used for design automation of SE droplets.

**Parameter**	**Parameter range & resolution**		
	Minimum	Maximum	Step-size
Orifice width	15	175	1.5
Aspect ratio	1	3	0.25
Normalized oil inlet	1	4	0.25
Normalized water inlet	1	4	0.25
Expansion ratio	1	6	0.25
Flow rate ratio	0.69	22	2
Capillary number	0.014	0.5	0.025

Supplementary Table 7: Comparison of DAFD 3.0 (current tool) and DAFD 1.0 (2021).

Metric	DAFD 3.0	DAFD 1.0
Droplet size/rate prediction	■	■
Droplet diameter range	15–250 μm	25–250 μm
Droplet generation rate range	12000 Hz	500 Hz
Diameter prediction error	5.4%	11.3%
Generation rate prediction error	16.6%	33.5%
As-yet-unseen fluids and geometries prediction error	10.1%	92.7%
Range of interfacial tension	0.31–29 mN/m	5 mN/m
Range of dispersed fluid viscosity	0.86–42 mPa·s	1 mPa·s
Range of continuous fluid viscosity	1.3–57 mPa·s	57 mPa·s
Oil-in-aqueous droplets	■	
Double emulsions size & rate prediction	■	
Fluid agnostic design automation	■	
Double emulsion design automation	■	
Predicts dimensionless droplet diameter	■	

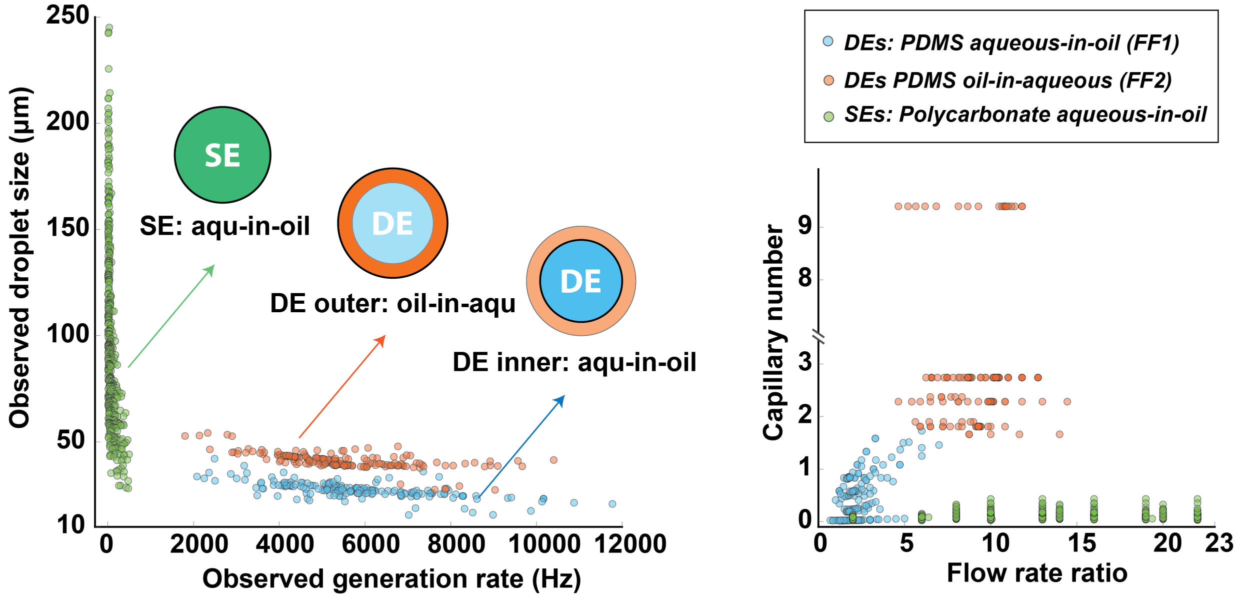

Supplementary Figure 1: Distribution of observed diameters and generation rates alongside the distribution of capillary numbers and flow rate ratios in the comprehensive dataset.

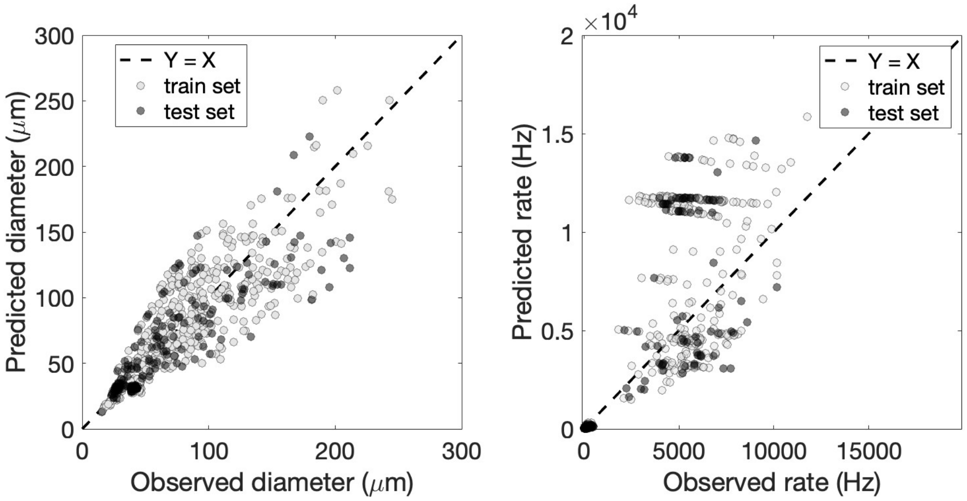

Supplementary Figure 2: A representative randomized training and testing run using the Fu at el. scaling law. See Supplementary Note 1 and Supplementary Table 2 for more information. A total of 15 randomized training/testing sessions were carried out.

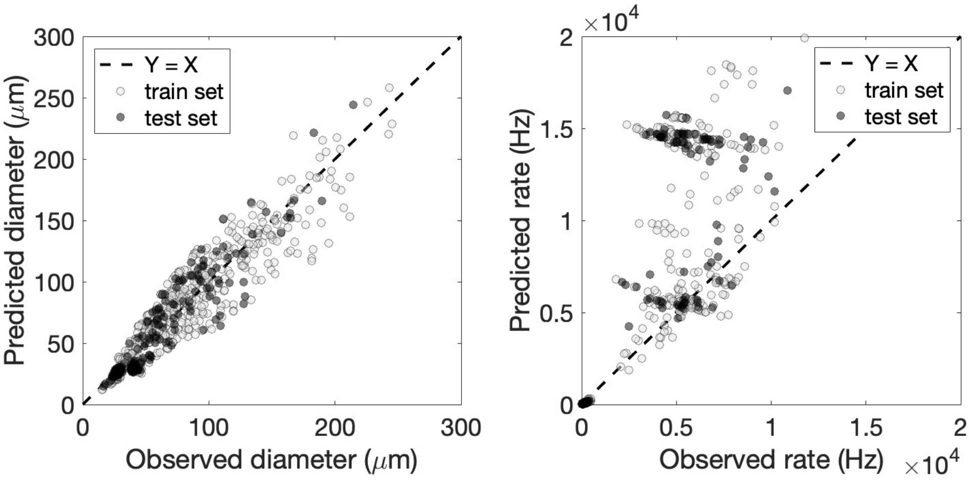

Supplementary Figure 3: A representative randomized training and testing run using the improved scaling law adapted from Fu et al. scaling law. See Supplementary Note 1 and Supplementary Table 2 for more information. A total of 15 randomized training/testing sessions were carried out.

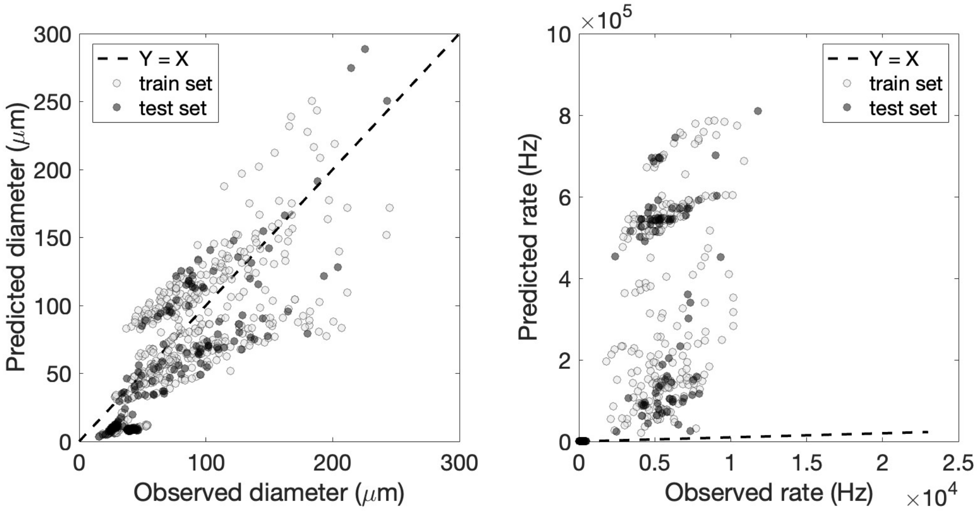

Supplementary Figure 4: A representative randomized training and testing run using the scaling law proposed by Liu et al. See Supplementary Note 1 and Supplementary Table 2 for more information. A total of 15 randomized training/testing sessions were carried out.

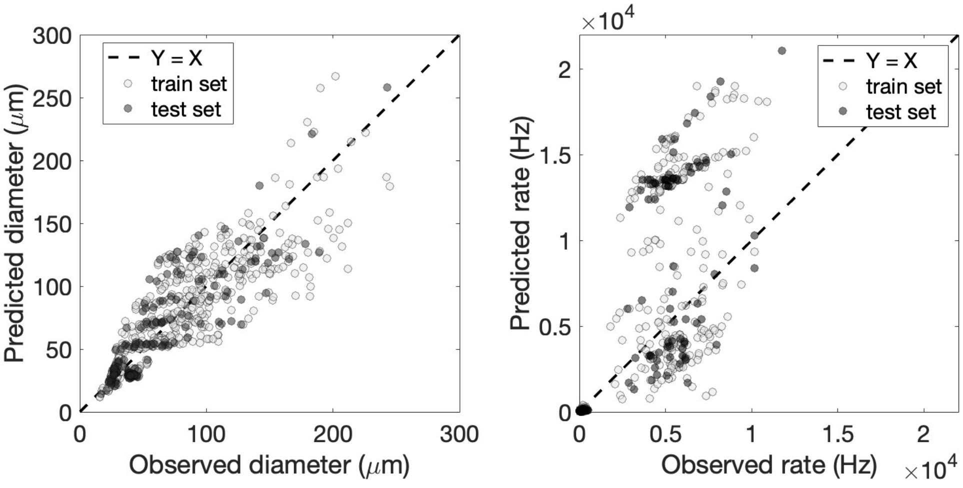

Supplementary Figure 5: A representative randomized training and testing run using the improved version of the scaling law originally proposed by Liu et al. See Supplementary Note 1 and Supplementary Table 2 for more information. A total of 15 randomized training/testing sessions were carried out.

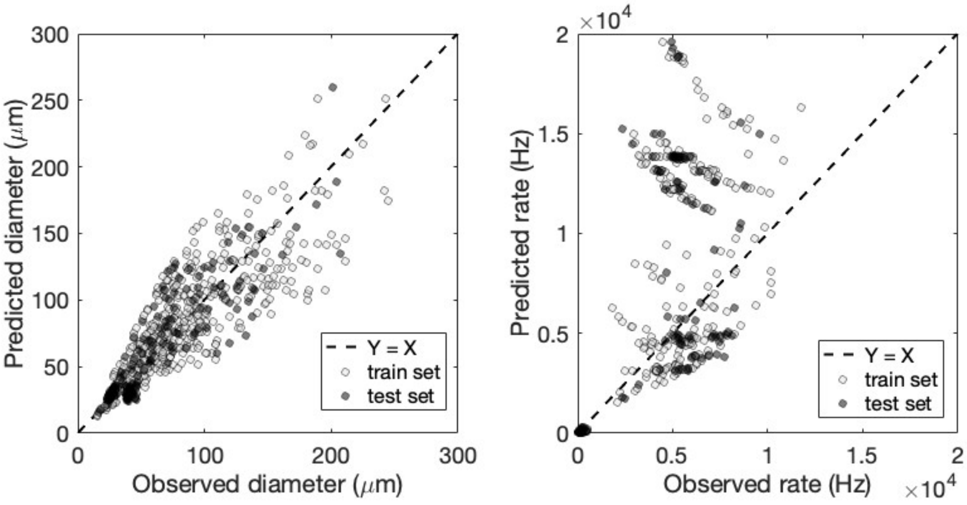

Supplementary Figure 6: A representative randomized training and testing run using the scaling law originally proposed by Xu et al. See Supplementary Note 1 and Supplementary Table 2 for more information. A total of 15 randomized training/testing sessions were carried out.

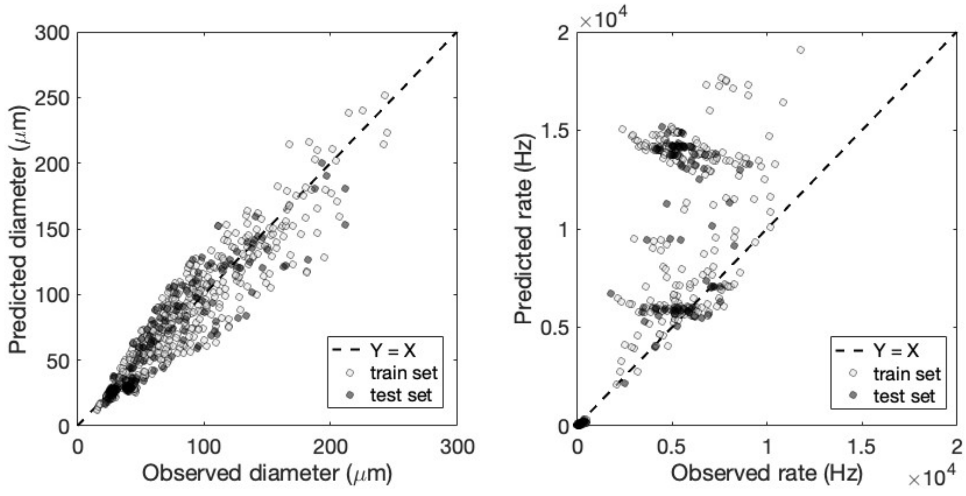

Supplementary Figure 7: A representative randomized training and testing run using the improved version of the scaling law originally proposed by Xu et al. See Supplementary Note 1 and Supplementary Table 2 for more information. A total of 15 randomized training/testing sessions were carried out.

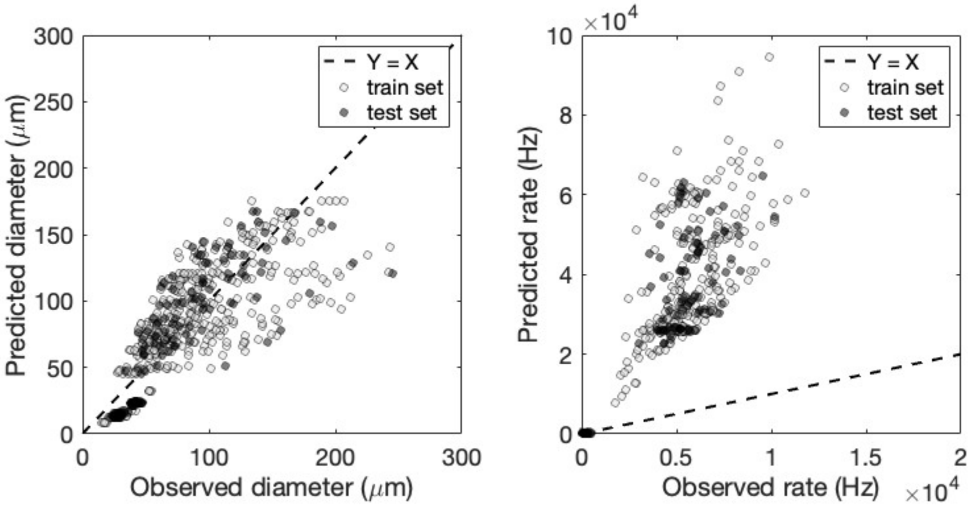

Supplementary Figure 8: A representative randomized training and testing run using the scaling law proposed by Lee et al. See Supplementary Note 1 and Supplementary Table 2 for more information. A total of 15 randomized training/testing sessions were carried out.

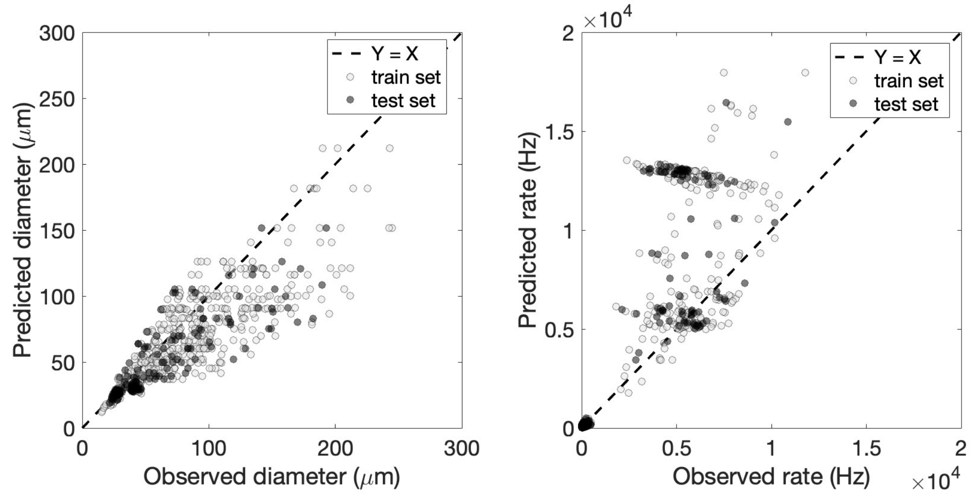

Supplementary Figure 9: A representative randomized training and testing run using the scaling law proposed by Ward et al. See Supplementary Note 1 and Supplementary Table 2 for more information. A total of 15 randomized training/testing sessions were carried out.

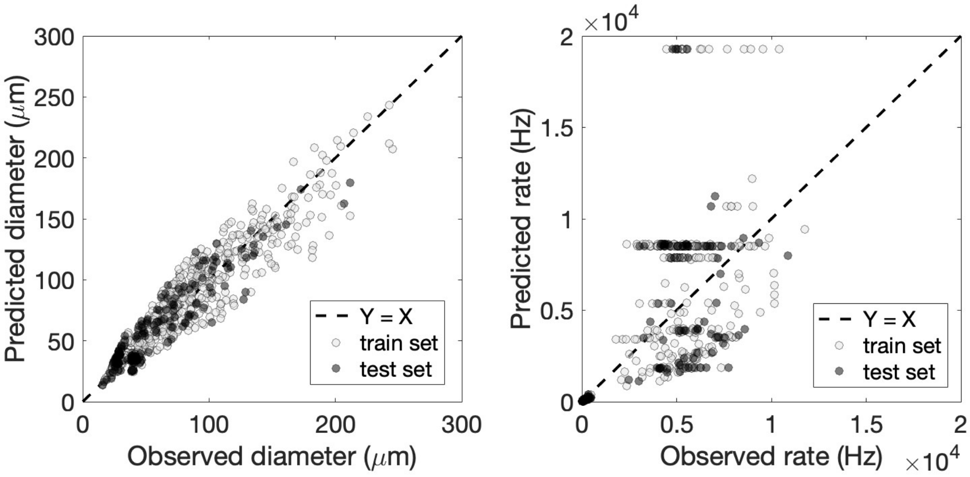

Supplementary Figure 10: A representative randomized training and testing run using the scaling law proposed by Zheya Liu et al. See Note 1 and Supplementary Table 2 for more information. A total of 15 randomized training/testing sessions were carried out.

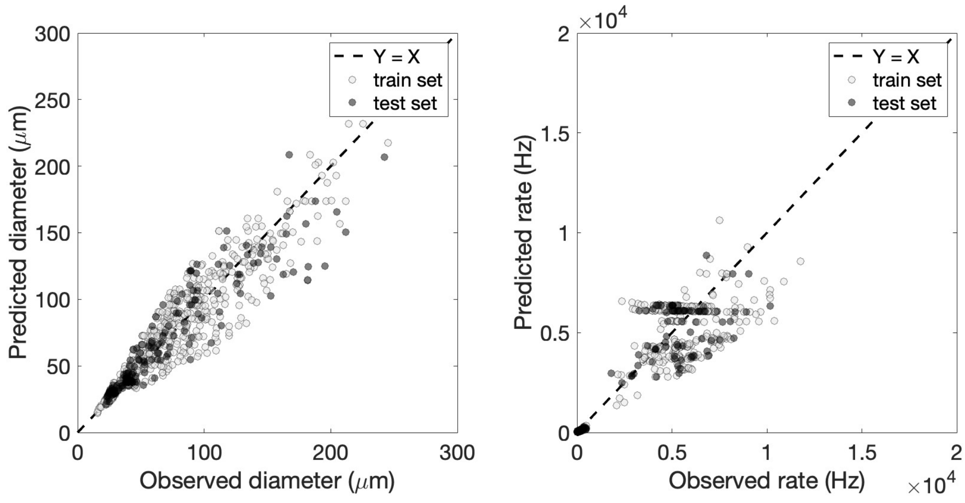

Supplementary Figure 11: A representative randomized training and testing run using the scaling law proposed by us. See Supplementary Note 1 and Supplementary Table 2 for more information. A total of 15 randomized training/testing sessions were carried out.

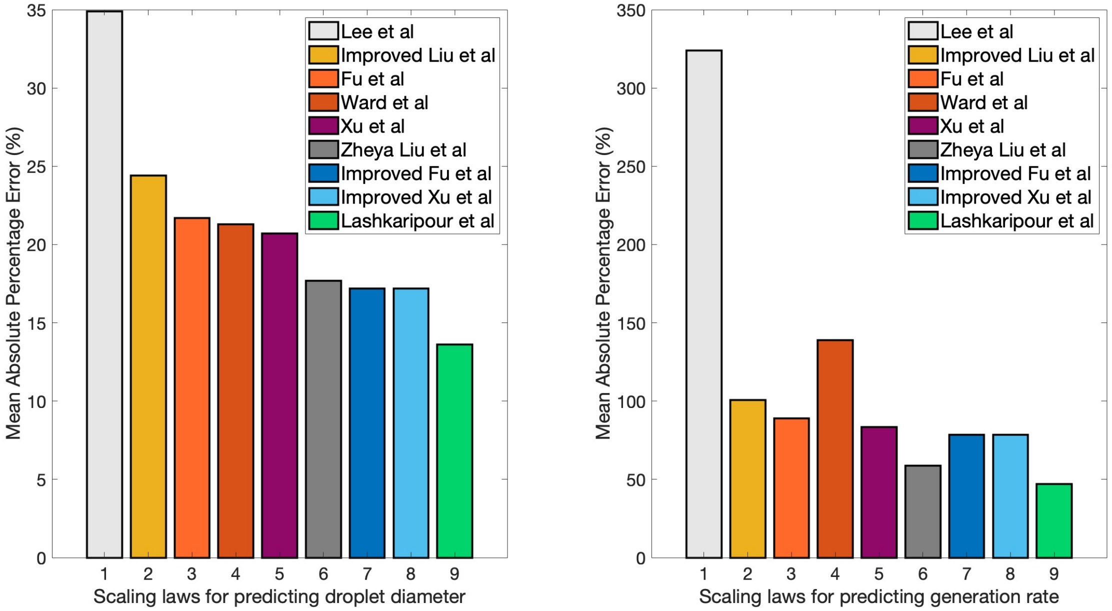

Supplementary Figure 12: Comparison of all the scaling laws in predicting droplet diameters and generation rates in our comprehensive dataset across 15 randomized replicates. Bars represent the mean values across 15 replicates. See Supplementary Note 1 and Supplementary Table 2 for more information.

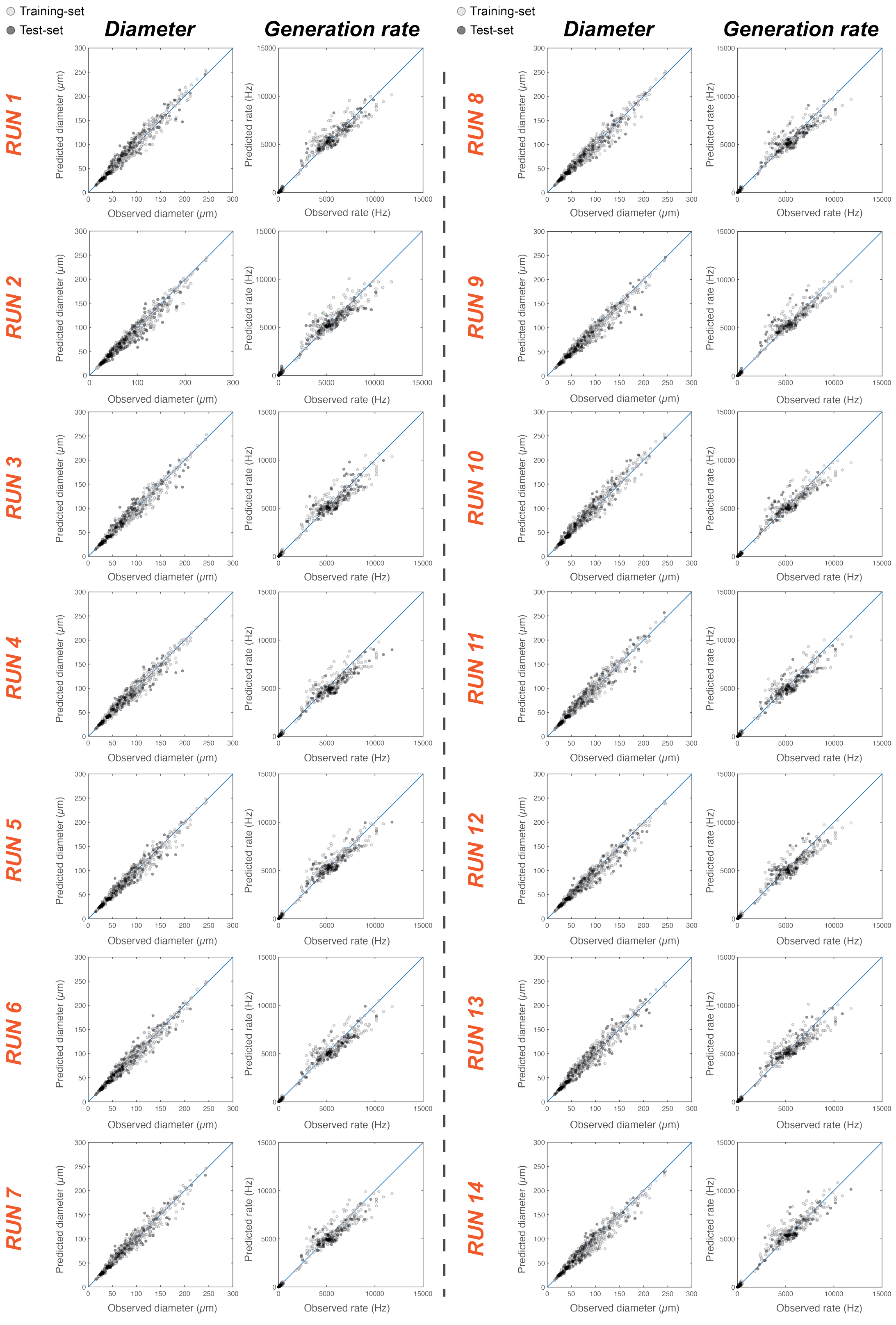

Supplementary Figure 13: Comparison of diameter and generation rate predictions of 14 random consecutive training runs for neural networks.

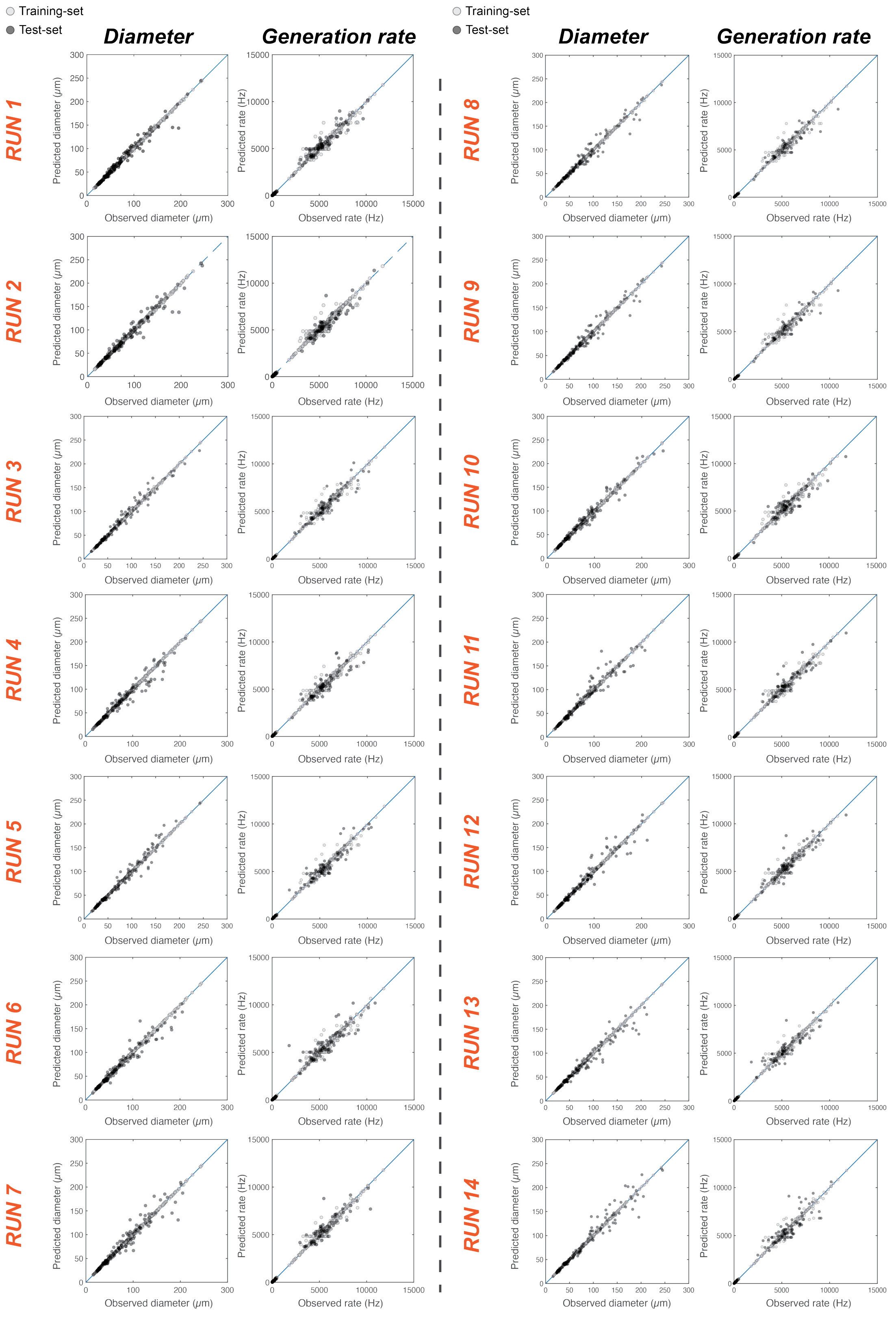

Supplementary Figure 14: Comparison of diameter and generation rate predictions of 14 random consecutive training runs for boosted decision trees.

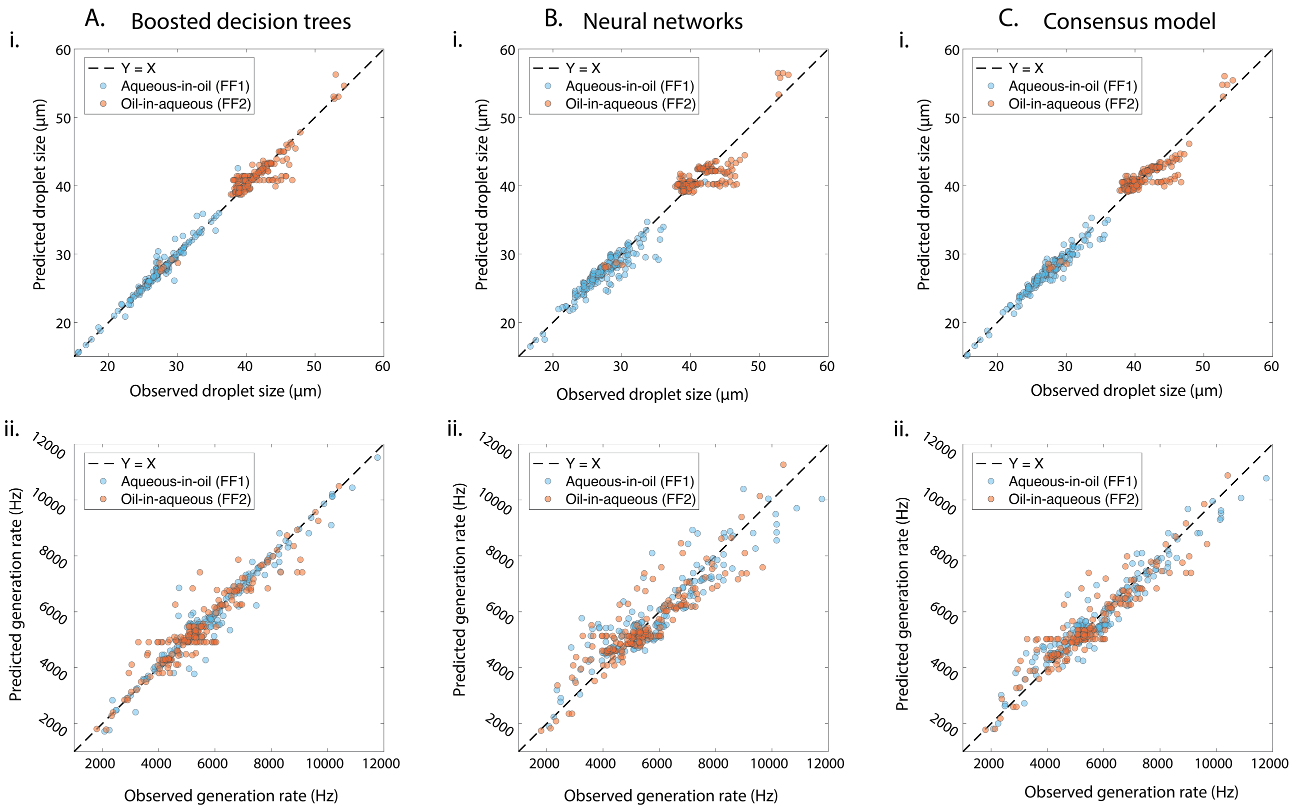

Supplementary Figure 15: A randomized and representative comparison of the trained machine learning models in predicting the inner and outer diameters of DEs in the comprehensive dataset.

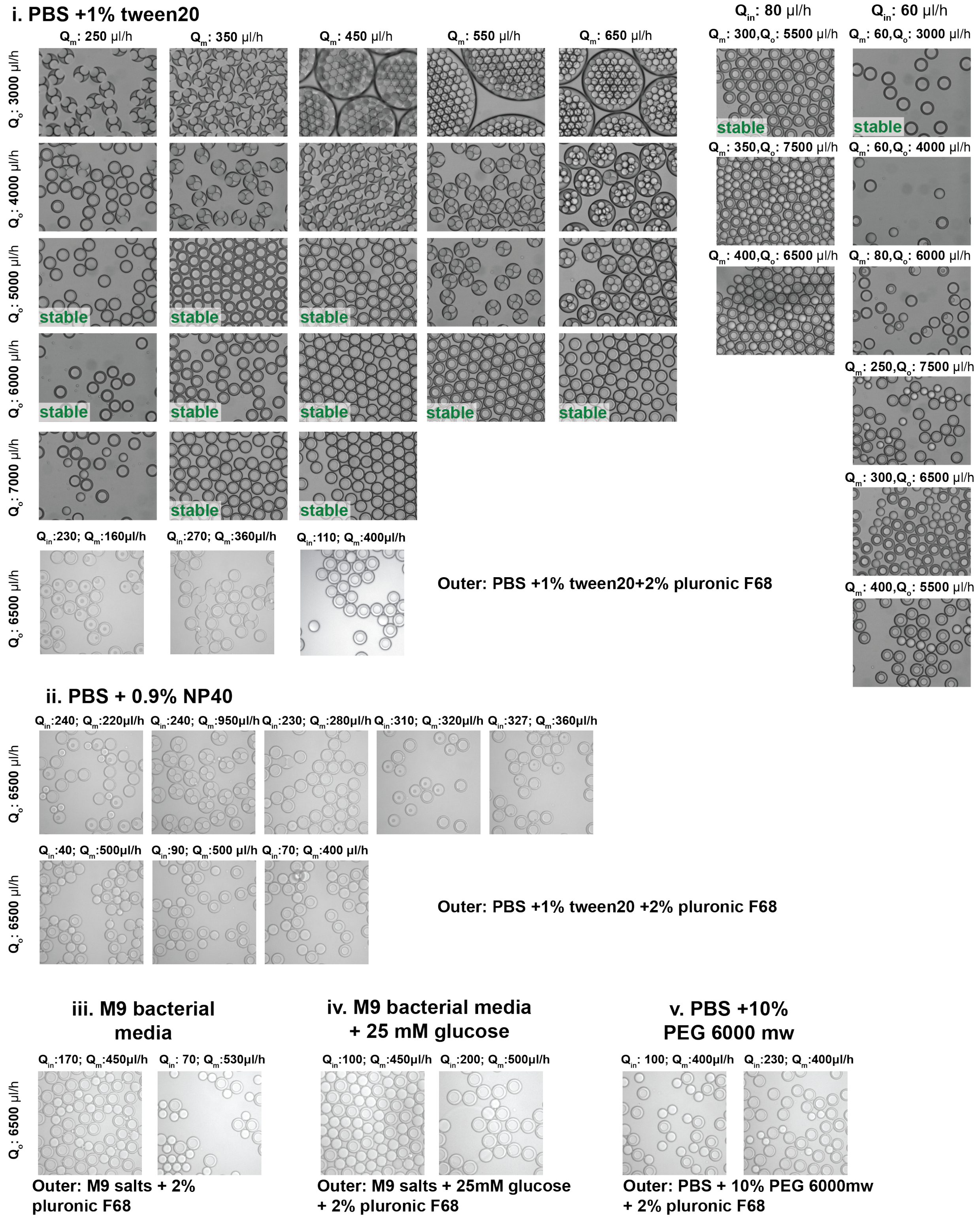

Supplementary Figure 16: Images for unstable DE droplets generated using 37 new conditions (5 different fluid combinations and varied inner, middle, and outer fluid flow rates). Experiments were carried out once.

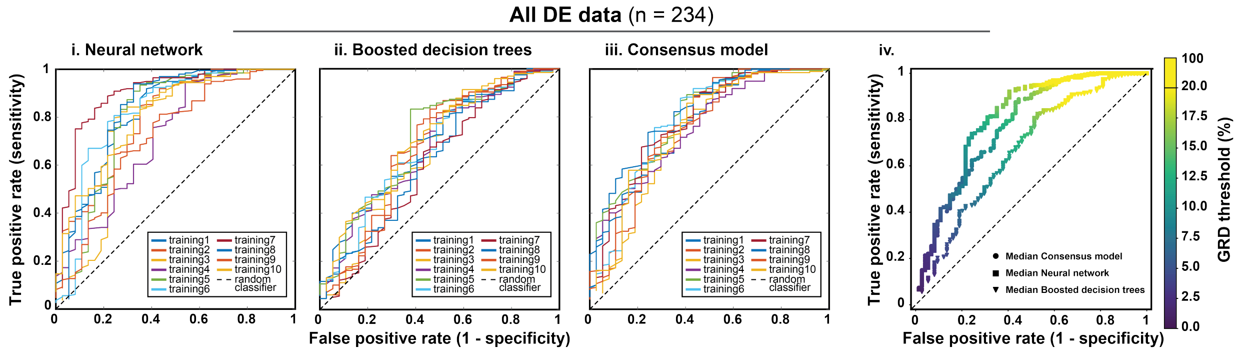

Supplementary Figure 17: Receiver operating characteristic (ROC) curves for predicting stable or unstable DE generation while varying GRD threshold for 10 consecutive and randomized training sessions for (i.) neural networks, (ii.) boosted decision trees, and (iii.) consensus models. True positive rate (sensitivity) indicates conditions predicted to yield stable DEs that generated stable DEs. False positive rate (1− specificity) indicates conditions predicted to yield stable DEs that generated multiple/missing core DEs. The dashed line represents a 1:1 line and indicates the performance of a random classifier. (iv.) Median values of true positive and false positive rates for 10 consecutive training sessions. Marker color indicate the threshold GRD value used to predict whether conditions would generate stable/unstable DEs.

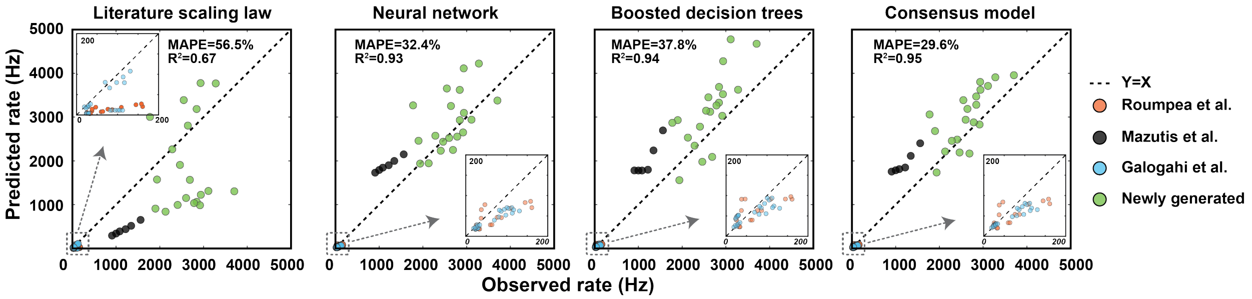

Supplementary Figure 18: A representative and randomized comparison of generalizability of literature scaling law with our different machine learning models in predicting generation rate of datasets with as-yet unseen fluids, geometries, and materials. Predictions were made for a total of 15 randomized replicates.

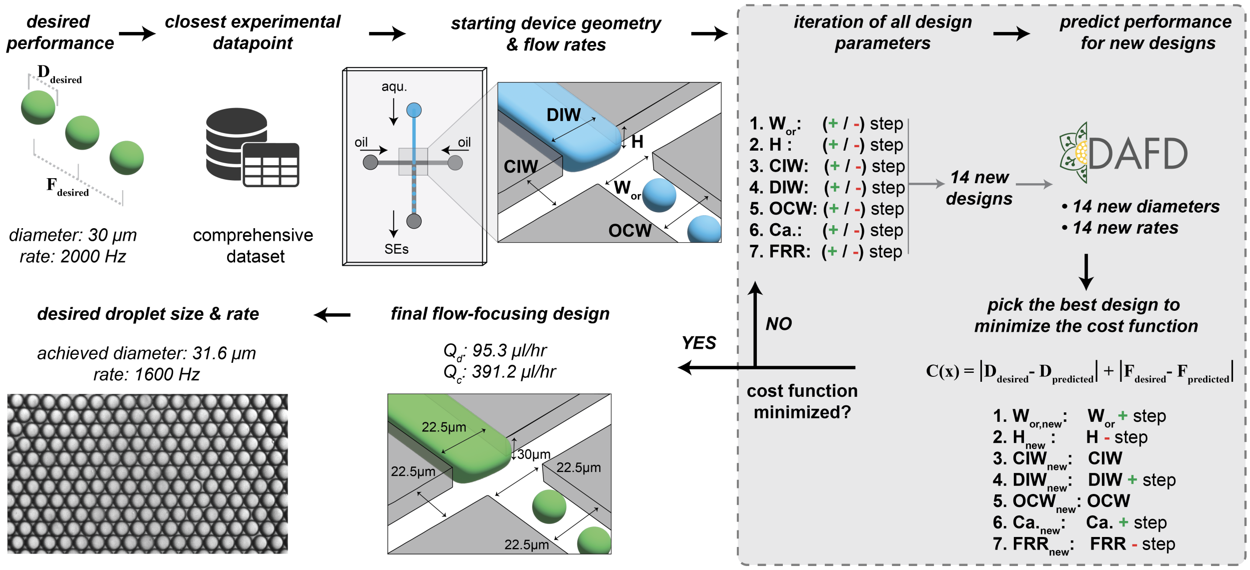

Supplementary Figure 19: The developed algorithm for design automation of single emulsions. Alongside a representative micrograph of single emulsions.

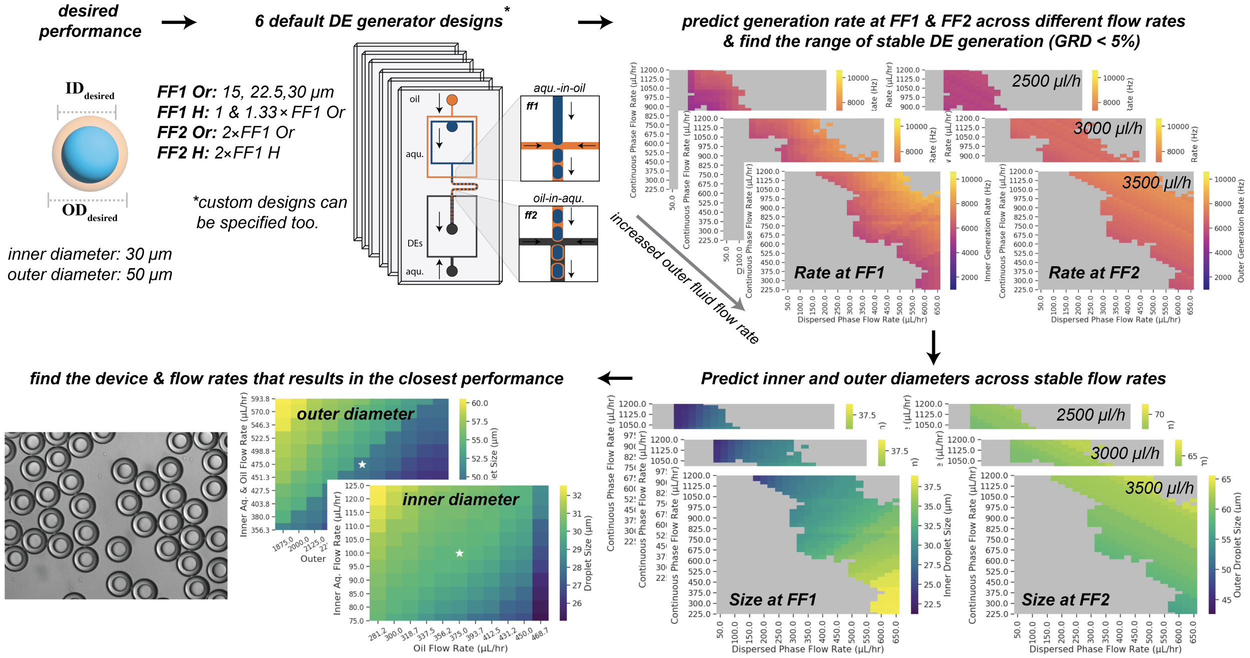

Supplementary Figure 20: The developed algorithm for design automation of double emulsions takes the desired inner and outer diameters and the fluid properties of the inner, middle, and outer fluids and finds the device geometry and flow rates that result in the user-specified desired performance. Alongside a representative micrograph of double emulsions.

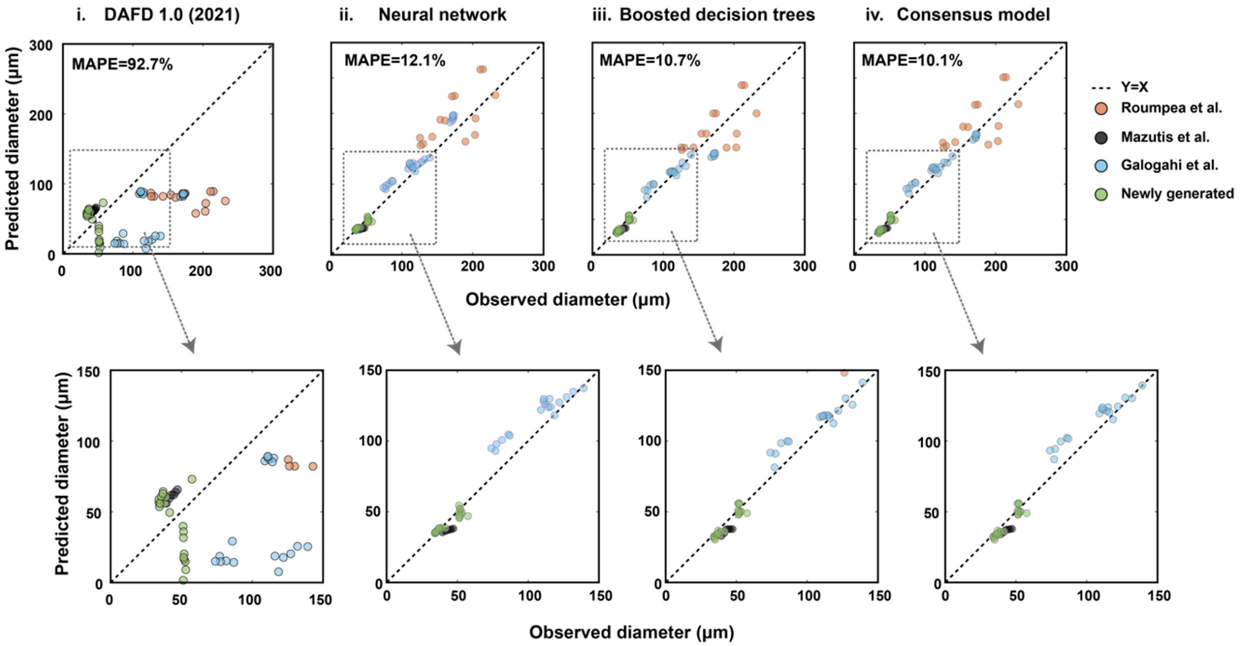

Supplementary Figure 21: Comparison of the generalizability of DAFD 1.0 [3] and DAFD 3.0 (current tool) to as-yet-unseen fluids and geometries. A representative randomized train/test session is shown. The training/testing session was carried out across 15 randomized replicates and average MAPEs are reported.

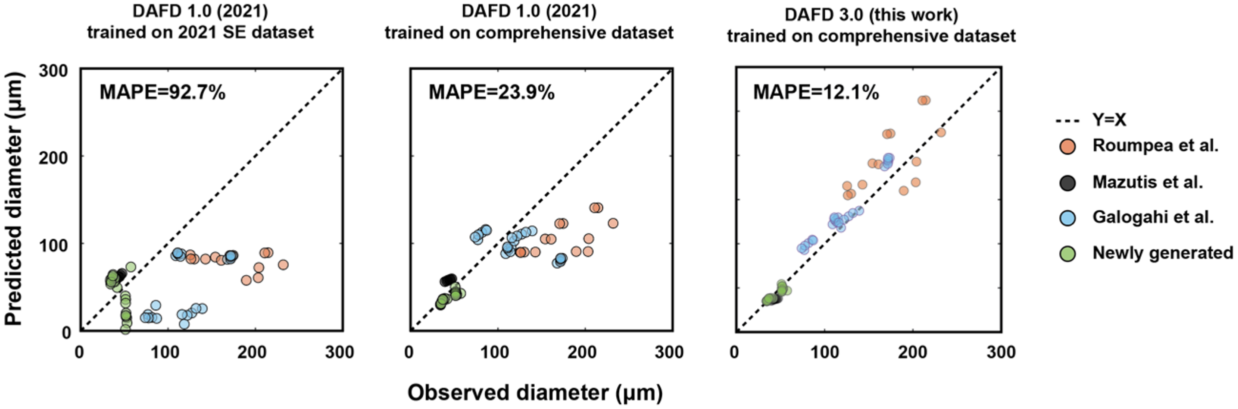

Supplementary Figure 22: Comparison of the generalizability of DAFD 1.0's neural network trained on SE dataset (2021), DAFD 1.0's neural network trained on the comprehensive dataset, and DAFD 3.0's neural network trained on the comprehensive dataset to as-yet-unseen fluids and geometries. A representative randomized train/test session is shown. The training/testing session was carried out across 15 randomized replicates and average MAPEs are reported.

Supplementary References

[1] Loïc Chagot, César Quilodrán-Casas, Maria Kalli, Nina M Kovalchuk, Mark JH Simmons, Omar K Matar, Rossella Arcucci, and Panagiota Angeli. Surfactant-laden droplet size prediction in a flow-focusing microchannel: a data-driven approach. *Lab on a Chip*, 22(20):3848–3859, 2022.

[2] Taotao Fu, Yining Wu, Youguang Ma, and Huai Z Li. Droplet formation and breakup dynamics in microfluidic flow-focusing devices: From dripping to jetting. *Chemical engineering science*, 84:207–217, 2012.

[3] Ali Lashkaripour, Christopher Rodriguez, Noushin Mehdipour, Rizki Mardian, David McIntyre, Luis Ortiz, Joshua Campbell, and Douglas Densmore. Machine learning enables design automation of microfluidic flow-focusing droplet generation. *Nature communications*, 12(1):25, 2021.

[4] Wingki Lee, Lynn M Walker, and Shelley L Anna. Role of geometry and fluid properties in droplet and thread formation processes in planar flow focusing. *Physics of Fluids*, 21(3):032103, 2009.

[5] Haihu Liu and Yonghao Zhang. Droplet formation in microfluidic cross-junctions. *Physics of Fluids*, 23(8):082101, 2011.

[6] Zheyu Liu, Maojie Chai, Xin Chen, Seyed Hossein Hejazi, and Yiqiang Li. Emulsification in a microfluidic flow-focusing device: Effect of the dispersed phase viscosity. *Fuel*, 283:119229, 2021.

[7] Thomas Ward, Magalie Faivre, Manouk Abkarian, and Howard A Stone. Microfluidic flow focusing: Drop size and scaling in pressure versus flow-rate-driven pumping. *Electrophoresis*, 26(19):3716–3724, 2005.

[8] Jian Hong Xu, SW Li, Jing Tan, and GS Luo. Correlations of droplet formation in t-junction microfluidic devices: from squeezing to dripping. *Microfluidics and Nanofluidics*, 5:711–717, 2008.
