# Supplementary material for: Design automation of microfluidic single and double emulsion droplets with machine learning
Source: Nat Commun. 2024 Jan 2;15:83. doi: 10.1038/s41467-023-44068-3 (PMC10761910; doi:10.1038/s41467-023-44068-3)
Supplement: Supplementary file 2 — Reporting Summary [file 41467_2023_44068_MOESM2_ESM.pdf]

nature portfolio

Corresponding author(s): Polly Fordyce

Last updated by author(s): Nov 18, 2023

# Reporting Summary

Nature Portfolio wishes to improve the reproducibility of the work that we publish. This form provides structure for consistency and transparency in reporting. For further information on Nature Portfolio policies, see our Editorial Policies and the Editorial Policy Checklist.

## Statistics

For all statistical analyses, confirm that the following items are present in the figure legend, table legend, main text, or Methods section.

| n/a | Confirmed | |
|---|---|---|
| ☐ | ☒ | The exact sample size (*n*) for each experimental group/condition, given as a discrete number and unit of measurement |
| ☐ | ☒ | A statement on whether measurements were taken from distinct samples or whether the same sample was measured repeatedly |
| ☒ | ☐ | The statistical test(s) used AND whether they are one- or two-sided<br>*Only common tests should be described solely by name; describe more complex techniques in the Methods section.* |
| ☒ | ☐ | A description of all covariates tested |
| ☐ | ☒ | A description of any assumptions or corrections, such as tests of normality and adjustment for multiple comparisons |
| ☐ | ☒ | A full description of the statistical parameters including central tendency (e.g. means) or other basic estimates (e.g. regression coefficient) AND variation (e.g. standard deviation) or associated estimates of uncertainty (e.g. confidence intervals) |
| ☒ | ☐ | For null hypothesis testing, the test statistic (e.g. *F*, *t*, *r*) with confidence intervals, effect sizes, degrees of freedom and *P* value noted<br>*Give P values as exact values whenever suitable.* |
| ☒ | ☐ | For Bayesian analysis, information on the choice of priors and Markov chain Monte Carlo settings |
| ☒ | ☐ | For hierarchical and complex designs, identification of the appropriate level for tests and full reporting of outcomes |
| ☒ | ☐ | Estimates of effect sizes (e.g. Cohen's *d*, Pearson's *r*), indicating how they were calculated |

*Our web collection on statistics for biologists contains articles on many of the points above.*

## Software and code

Policy information about availability of computer code

| | |
|---|---|
| Data collection | All source code is written in Python 3.10.9 and Spyder 5.4.1 interface and is publicly available to all on an OSF repository: https://osf.io/938rs/ . The source code for DAFD is also available on our GitHub repository: https://github.com/CIDARLAB/dafd-website. Matlab 2023a was used to plot the graphs and Adobe illustrator 2023 was used to create the figures. |
| Data analysis | All data analysis was done using Python 3.10.9 using Spyder 5.4.1 interface and Matlab 2023a. The source code is publicly available on our GitHub repository: https://github.com/CIDARLAB/dafd-website and all data are available on an OSF repository: https://osf.io/938rs/. |

For manuscripts utilizing custom algorithms or software that are central to the research but not yet described in published literature, software must be made available to editors and reviewers. We strongly encourage code deposition in a community repository (e.g. GitHub). See the Nature Portfolio guidelines for submitting code & software for further information.

## Data

Policy information about availability of data

All manuscripts must include a data availability statement. This statement should provide the following information, where applicable:

- Accession codes, unique identifiers, or web links for publicly available datasets
- A description of any restrictions on data availability
- For clinical datasets or third party data, please ensure that the statement adheres to our policy

The comprehensive dataset created and curated in this study and its subsets (SE and DE datasets) have been deposited in the Open Science Framework (OSF) repository and are available at: https://osf.io/938rs/ and DAFD's website at: http://dafdcad.org

## Research involving human participants, their data, or biological material

Policy information about studies with human participants or human data. See also policy information about sex, gender (identity/presentation), and sexual orientation and race, ethnicity and racism.

| | |
|---|---|
| Reporting on sex and gender | N/A |
| Reporting on race, ethnicity, or other socially relevant groupings | N/A |
| Population characteristics | N/A |
| Recruitment | N/A |
| Ethics oversight | N/A |

Note that full information on the approval of the study protocol must also be provided in the manuscript.

# Field-specific reporting

Please select the one below that is the best fit for your research. If you are not sure, read the appropriate sections before making your selection.

☒ Life sciences ☐ Behavioural & social sciences ☐ Ecological, evolutionary & environmental sciences

For a reference copy of the document with all sections, see nature.com/documents/nr-reporting-summary-flat.pdf

# Life sciences study design

All studies must disclose on these points even when the disclosure is negative.

| | |
|---|---|
| Sample size | 868 datapoints were used to train machine learning models. This dataset is a curated combination of two previously published datasets of lashkaripour et al. Nature Communications 2021 (474 datapoints) and Calhoun et al. Lab on a Chip 2022 (394 or 197*2 datapoints on inner and outer diameters). 44 datapoints from previously published studies and 20 newly generated datapoints were used to assess the generalizability of the machine learning models. |
| Data exclusions | No data points were excluded. |
| Replication | All performance metrics were reported on 15 randomized training sessions. All replicates were successful. |
| Randomization | The train-set (80%) and test-set (20%) split was randomly initialized. All performance metrics were reported on 15 randomized training sessions. |
| Blinding | Our models were blinded to the 20% of the initial datasets for training. Also they were blinded to all datapoints used for assessing their generalizability. |

# Reporting for specific materials, systems and methods

We require information from authors about some types of materials, experimental systems and methods used in many studies. Here, indicate whether each material, system or method listed is relevant to your study. If you are not sure if a list item applies to your research, read the appropriate section before selecting a response.

## Materials & experimental systems

| n/a | Involved in the study | |
|---|---|---|
| x | | Antibodies |
| x | | Eukaryotic cell lines |
| x | | Palaeontology and archaeology |
| x | | Animals and other organisms |
| x | | Clinical data |
| x | | Dual use research of concern |
| x | | Plants |

## Methods

| n/a | Involved in the study | |
|---|---|---|
| x | | ChIP-seq |
| x | | Flow cytometry |
| x | | MRI-based neuroimaging |

## Plants

| | |
|---|---|
| Seed stocks | N/A. |
| Novel plant genotypes | N/A |
| Authentication | N/A |
